# Dynamic compensation, parameter identifiability, and equivariances

**DOI:** 10.1371/journal.pcbi.1005447

**Published:** 2017-04-06

**Authors:** Eduardo D. Sontag

**Affiliations:** Department of Mathematics and Center for Quantitative Biology, Hill Center, Rutgers University, Piscataway, New Jersey, United States of America; University of California Irvine, UNITED STATES

## Abstract

A recent paper by Karin et al. introduced a mathematical notion called dynamical compensation (DC) of biological circuits. DC was shown to play an important role in glucose homeostasis as well as other key physiological regulatory mechanisms. Karin et al. went on to provide a sufficient condition to test whether a given system has the DC property. Here, we show how DC can be formulated in terms of a well-known concept in systems biology, statistics, and control theory—that of parameter structural non-identifiability. Viewing DC as a parameter identification problem enables one to take advantage of powerful theoretical and computational tools to test a system for DC. We obtain as a special case the sufficient criterion discussed by Karin et al. We also draw connections to system equivalence and to the fold-change detection property.

## Author summary

A recently introduced mathematical notion called dynamical compensation of biological circuits was shown to play an important role in glucose homeostasis and other key physiological regulatory mechanisms. This paper explains how dynamical compensation can be formulated in terms of a well-known concept in systems biology, statistics, and control theory—that of parameter structural non-identifiability. Viewing dynamical compensation as a parameter identification problem enables one to take advantage of powerful theoretical and computational tools to test a system for dynamical compensation. As a special case, one obtains the sufficient criterion for dynamical compensation. The paper also draws connections to system equivalence and to the fold-change detection property. The non-identifiability characterization brings up an interesting contrast in the way in which one thinks of these properties in the two fields. From the point of view of robustness of behavior, one wishes that parameters do not influence much the response of a system. On the other hand, from the systems and parameter identification point of view, the more that a parameter affects behavior, the easier it is to estimate it, and poor sensitivity is taken as an indication of a poorly parametrized model.

## Introduction

The recent paper [1] argued that physiological control systems should ensure a precise dynamical response despite variations in certain parameters, lest pathological conditions arise. The authors highlighted the biological significance of this robustness property through the analysis of several biological systems, including models of plasma glucose response in the face of changes in insulin sensitivity, parathyroid hormone control of calcium homeostasis, and arterial oxygen regulation in response to hypoxia. They formally introduced the system property of *dynamical compensation (DC)* with respect to variations in a parameter *p*, meaning that the complete output dynamics is exactly the same, for any time dependent input, independently of the precise value of *p*. They went on to provide a sufficient condition for DC to hold, and applied this condition to verify that their physiological examples have the DC property.

In this paper, we frame the notion of DC in the context of two equivalent notions in systems biology: equivariances and parameter (non-)identifiability. We provide a necessary and sufficient condition for DC in the language of equivariances and partial differential equations, for which the sufficient condition given in [1] becomes a particular case, as does the condition given in [2] for fold-change detection (FCD) or input symmetry invariance, a Weber-like law in psychophysics [3]. We also provide alternative necessary and sufficient conditions for DC, using some of the rich tools in the existing literature on systems identification and nonlinear control theory, for which effective algorithms are available. The non-identifiability characterization brings up an interesting contrast in the way in which one thinks of these properties in the two fields. From the point of view of robustness of behavior, one wishes that parameters do not influence much the response of a system. On the other hand, from the systems and parameter identification (“reverse engineering”) point of view, the more that a parameter affects behavior, the easier it is to estimate it, and poor sensitivity is taken as an indication of a poorly parametrized model.

We focus our paper on a self-contained discussion, with examples, of the fact that DC is equivalent to other known concepts. There is, in addition, an extensive literature on parameter identifiability, which may be consulted for more details, mathematical theory, and effective algorithms based on computer algebra methods [4, 5, 6, 7, 8, 9, 10, 11, 12, 13, 14, 15, 16].

This paper is organized as follows. After formally setting up the questions to be studied, we present several characterizations: equivariances, Lie derivatives, and derivatives of outputs at the initial time. We then re-visit the examples from [1] in view of these characterizations, working out each example. A Discussion section summarizes the results, compares methodologies, and mentions weaker versions of DC. The Methods section discusses the proofs of the various results.

### Systems and equivalence

We formally state definitions in the language of dynamical systems with inputs and outputs, the standard paradigm in control systems theory [17]:

$$\dot{x}=f(x,u,p)\;,\;y=h\left(x,u,p\right)\;,\;x\left(0\right)=\xi_{p}\;,$$ (1)

or, more explicitly,

$$\frac{dx}{dt}\left(t\right)=f(x\left(t\right),u\left(t\right),p)\;,\;y\left(t\right)=h\left(x\left(t\right),u\left(t\right),p\right)\;,\;\;t\geq 0\;,\;x\left(0\right)=\xi_{p}.$$

The functions *f*, *h* describe respectively the dynamics and the read-out map; *u* = *u*(*t*) is an input (stimulus, excitation) function, assumed to be piecewise continuous in time, *x*(*t*) is an *n*-dimensional vector of state variables, *y*(*t*) is the output (response, reporter) variable, and *p* is the parameter (or vector of parameters) that we wish to focus our attention on. In typical applications, *y*(*t*) = *x*_*i*_(*t*) is a coordinate of *x*. Values of states, inputs, outputs, and parameters are constrained to lie in particular subsets X, U, Y, P respectively, of Euclidean spaces $R^{n},R^{m},R^{q},R^{s}$. Typically in biological applications, one picks X as the set of positive vectors in $R^{n}$, *x* = (*x*_1_, *x*_2_, …, *x*_*n*_) such that *x*_*i*_ > 0 for all *i*, and similarly for the remaining spaces. The state $\xi_{p}\in X$ is an initial state, which could be different for different parameters; thus we view *ξ*_*p*_ as a function P→X. (We prefer the notation *ξ*_*p*_ instead of *ξ*(*p*), so that we do not confuse *p* with the time variable.) For each input u:[0,∞)→U, we write the solution of Eq (1) with an initial condition *x*(0) = *ξ* (for example, *x*(0) = *ξ*_*p*_) as

x(t)=φ(t,ξ,u,p)

and the corresponding system response, which is obtained by evaluating the read-out map along trajectories, as

y(t)=h(φ(t,ξ,u,p),u(t),p)=ψ(t,ξ,u,p).

We assume that for each input and initial state (and each fixed parameter *p*), there is a unique solution of the initial-value problem $\dot{x}=f(x,u,p)$, *x*(0) = *ξ*, so that the mapping *φ* is well-defined; see [17] for more discussion, regularity of *f*, properties of ODE’s, global existence of solutions, etc. We refer to Eq (1) as a *parametrized* (because of the explicit parameter) *initialized* (because of the specification of a given initial state for each parameter) family of systems.

Depending on the application, one might wish to impose additional restrictions on *ξ*_*p*_. For example, in [1] an additional requirement is that 0∈U and $\xi_{p}\in X$ is an equilibrium when *u*(0) = 0, which translates into *f*(*ξ*_*p*_, 0, *p*) = 0, and a similar requirement is made in [2] (this latter reference imposes the requirement that for each constant input *u*(*t*) ≡ *μ* there should exist an equilibrium, in fact). One may also impose stability requirements on this equilibrium; nothing changes in the results to be stated.

The “dynamic compensation” property in [1]—we prefer to use the terminology P-*invariance*—is the property that outputs should be always the same, independent of the particular parameter. Formally:

$$\psi \left(t,\xi_{p},u,p\right)\;=\;\psi \left(t,\xi_{q},u,q\right)\;\;\forall \;t\geq 0,\;u\left(\cdot \right),\;p,\;q\;.$$ (2)

The question addressed in [1] was that of providing conditions on the functions *f*, *h*, and *ξ* so that Property Eq (2) holds.

## Results

We start by re-stating P-invariance in a more convenient form. Let us fix from now on an arbitrary p∈P, which we write as “1” (for example, if P is the set of all positive vectors (*p*_1_, …, *p*_*s*_), 1 could naturally be taken as the vector (1, 1, …, 1)). Let us write *f*(*x*, *u*) ≔ *f*(*x*, *u*, 1), *h*(*x*, *u*) ≔ *h*(*x*, *u*, 1), *ξ* ≔ *ξ*_1_, *φ*(*t*, *ξ*, *u*) ≔ *φ*(*t*, *ξ*, *u*, 1), and *ψ*(*t*, *ξ*, *u*) ≔ *ψ*(*t*, *ξ*, *u*, 1).

In control theory, one says that, for any given parameter *p*, the system (1) is said to be *input/output* (I/O) *equivalent* to the system

$$\dot{x}=f(x,u)\;,\;y=h\left(x,u\right)\;,\;x\left(0\right)=\xi \;.$$ (3)

provided that

$$\psi \left(t,\xi_{p},u,p\right)\;=\;\psi \left(t,\xi ,u\right)\;\;\forall \;t\geq 0,\;u\left(\cdot \right)\;.$$ (4)

Therefore, Eq (2) is equivalent to the property that, for every parameter *p*, Eq (1) should be I/O equivalent to Eq (3). Thus, the P-invariance property is equivalent to the property that the parameters *p* are (structurally) *unidentifiable* from input/output data, a notion which was originally introduced by Bellman and Astrom in the 1970s [4]. Two approaches to testing I/O equivalence (or its lack) are described next.

### Equivariances

**Definition 1** *Given a parametrized initialized family of* system (1), *a set of differentiable mappings*

$${\left\{\rho_{p}:X\to X\right\}}_{p\in P}$$

*is an* equivariance family *provided that:*

$$f(\rho_{p}(x),\;u,\;p)\;=\;{\left(\rho_{p}\right)}_{*}(x)f(x,\;u)$$ (5a)

$$h(\rho_{p}(x),\;u,\;p)\;=\;h(x,\;u)$$ (5b)

$$\rho_{p}(\xi )\;=\;\xi_{p}$$ (5c)

*for all* x∈X, u∈U, *and* p∈P, *where in general* ρ_*_ *denotes the Jacobian matrix of a transformation* *ρ*.

An important observation is as follows.

**Proposition 1** *If an equivariance family exists*, *then the parametrized family is* P-*invariant.*

Observe that Eq (5a) is a first order quasilinear partial differential equation on the components of the vector function *ρ*_*p*_ (for each constant value *u* ∈ *U* and each parameter p∈P), subject to the algebraic constraints given by Eqs (5b) and (5c). Such equations are usually solved using the method of characteristics [18]. In principle, testing for existence of a solution through a “certificate” such as an equivariance is far simpler than testing all possible time-varying inputs *u*(*t*) in the definition of equivalence, in a fashion analogous to the use of Lyapunov functions for testing stability or of value functions in the Hamilton-Jacobi-Bellman formulation of optimal control theory [17]. The paper [2] discusses the relation to classical equivariances in actions of Lie groups and symmetry analysis of nonlinear dynamical systems. Proposition 1 is proved in the Methods section.

It is a deeper result that the existence of an equivariance is also *necessary* as well as sufficient. This converse result, as some of the other converses to be mentioned, requires that the vector fields *f*(⋅, *u*), for each constant *u*, as well as the output function *h*, be real-analytic, that is, that they can be expanded into locally convergent power series. The expressions that commonly appear in systems biology models, such as mass action kinetics (polynomial functions) or Hill functions (rational functions) are real analytic, as are trigonometric, logarithmic, and exponential functions, so analyticity is not a strong restriction on the theory. An additional technical assumption, verified by all reasonable models including all examples in this paper, is that the systems are *irreducible*, meaning accessible from the initial state and observable. These notions are reviewed in the Methods section. Intuitively, accessibility means that no conservation laws restrict motions to proper submanifolds. For analytic systems, accessibility is equivalent to the property that the set of points reachable from *ξ* has a nonempty interior. Intuitively, observability means that no pairs of distinct states can give rise to an identical temporal response to all possible inputs.

**Proposition 2** *If the parametrized family is* P-*invariant and the systems are irreducible for each* *p*, *then an equivariance family exists.*

Necessity tells us that it is always worth searching for an equivariance, when attempting to prove that a system has P-invariance. Often one can guess such functions by looking at the structure of the equations. Proposition 2 is proved in the Methods section.

### Identifiability and Lie derivatives

For simplicity, we now restrict attention to systems in which inputs appear linearly (or more precisely, affinely); that is, systems defined by differential equations of the following general form:

$$\dot{x}\;=\;g_{0}\left(x,p\right)+u_{1}g_{1}\left(x,p\right)+\ldots +u_{m}g_{m}\left(x,p\right)\;,\;y=h\left(x,p\right)\;,\;x\left(0\right)=\xi_{p}\;,$$ (6)

where we assume that the input-value set U is a convex subset of $R^{m}$. Linearity in inputs is not a serious restriction, nor is not having an explicit input in the read-out map, and it is easy to generalize to more general classes of systems, but the notations become far less elegant. We write the *s* coordinates of *h* as *h* = (*h*_1_, …, *h*_*s*_).

Consider the following set of functions (“elementary observables” of the system):

$$H\;≔\;\left\{\left.L_{g_{i_{1}}}\;\ldots \;L_{g_{i_{k}}}h_{j}\;\right|\;\left(i_{1},\ldots ,i_{k}\right)\in {\left\{0,\ldots ,m\right\}}^{k},\;k\geq 0,\;j\in \left\{1,\ldots ,s\right\}\right\}\;.$$

We are using the notation

$$L_{X}H\;≔\;\nabla H\cdot X$$

for the directional or Lie derivative of the function *H* with respect to the vector field *X*, and an expression as *L*_*Y*_*L*_*X*_*H* means an iteration *L*_*Y*_ (*L*_*X*_*H*). We include in particular the case in which *k* = 0, in which case the expression in the defining formula is simply *h*_*j*_. For example, if the system is

$${\dot{x}}_{1}=\alpha u-\delta x_{1}\;,\;{\dot{x}}_{2}=\beta u-\gamma x_{1}x_{2}\;,\;y=x_{2}$$

(initial states do not matter at this point; the parameters of interest could be all or a subset of *α*, *β*, *γ*, *δ*), then

$$g_{0}\;=\;\left(\begin{array}{c} -\delta x_{1}\\ -\gamma x_{1}x_{2} \end{array}\right),\;\;g_{1}=\;\left(\begin{array}{c} \alpha \\ \beta \end{array}\right),\;\;h\left(x\right)=x_{2}$$

and we compute, for example,

$$L_{g_{0}}h\;=\;\left(0\;1\right)\left(\begin{array}{c} -\delta x_{1}\\ -\gamma x_{1}x_{2} \end{array}\right)\;=\;-\gamma x_{1}x_{2},$$

$$L_{g_{1}}L_{g_{0}}h\;=\;\left(-\gamma x_{2}\;\;-\gamma x_{1}\right)(\begin{array}{c} \alpha \\ \beta \end{array})\;=\;-\alpha \gamma x_{2}-\beta \gamma x_{1}\;.$$

A necessary condition for invariance is as follows.

**Proposition 3** *If a system is* P-*invariant*, *then all elements in* H, *when evaluated at the initial state*, *are independent of the values of parameters* *p*.

The Methods section discusses a proof of this Proposition. This condition is most useful when proving *non*-invariance. One simply finds one element of H which depends on parameters. This test can also be used as a test for identifiability, that is to say the possibility of recovering all parameters perfectly from outputs: if one can reconstruct the values of *p* from the elementary observables (evaluated at the initial state), that is to say if the map

$$p\;\mapsto \;{\left(L_{g_{i_{1}}}\;\ldots \;L_{g_{i_{k}}}h_{j}\left(\xi_{p}\right)\right)}_{\left(i_{1},\ldots ,i_{k}\right)\in {\left\{0,\ldots ,m\right\}}^{k},\;k\geq 0,\;j\in \left\{1,\ldots ,s\right\}}$$

is one-to-one, then the parameters are identifiable. We discuss two examples in the section “Examples from the paper [1]”.

There is a converse of this property, under the additional assumption of analyticity.

**Proposition 4** *If the system is analytic and if all elements in* H, *when evaluated at the initial state*, *are independent of the values of parameters* *p*, *then the system is* P-*invariant*.

An interpretation of this result in terms of identifiability is as follows: if one can recover all parameters as functions (perhaps nonlinear) of the elementary observables, then there is complete parameter identifiability (two different parameters give two different outputs, at least for some input). In other words, if the elementary observables give the same values for some two specific parameters p,q∈P, then the output for any possible inputs, whether step inputs or arbitrary inputs, is the same for these two parameters. See the Methods section for a discussion of this converse statement.

### Identifiability and time derivatives at *t* = 0

Suppose that an input *u*(*t*) is infinitely differentiable on *t*. Then, and assuming that *f* and *h* are infinitely differentiable on their arguments, the corresponding output *y*(*t*), for any initial state, will also be infinitely differentiable (see e.g. the differential equations appendix in [17]). We assume now that the system is linear in controls, that is, that the equations have the form in Eq (6). It follows by iterating inputs and using the chain rule that the *i*th derivative *y*^(*i*)^ of the output (*i* > 0) is a polynomial on the first *i* − 1 derivatives of the inputs. For example, $y^{\prime }=L_{g_{0}}h\;+\;u_{1}L_{g_{1}}h\;+\;\ldots \;+\;u_{m}L_{g_{m}}h$ and (taking for simplicity *m* = 1)

$$y^{\prime \prime }=L_{g_{0}}^{2}h+u_{1}\left(L_{g_{0}}L_{g_{1}}h+L_{g_{1}}L_{g_{0}}h\right)+u_{1}^{2}L_{g_{1}}^{2}h+u_{1}^{\prime }L_{g_{1}}h\;.$$

Now let us consider the derivatives *m*_*i*_ ≔ *y*^(*i*)^(0^+^). The *m*_*i*_’s are independent of parameters if and only if the coefficients of these polynomials on the inputs and their derivatives, which are themselves linear combinations of Lie derivatives, are independent of parameters.

**Proposition 5** *If a system is* P-*invariant*, *then all the derivatives* *m*_*i*_ *are independent of the values of parameters* *p*.

A converse holds as well.

**Proposition 6** *If a system is analytic and if all the derivatives* *m*_*i*_ *are independent of the values of parameters* *p*, *then the system is* P-*invariant*.

Typically, all higher-order derivatives of outputs can be expressed as a function of a finite number of derivatives, which helps make computations effective using computer algebra packages. The theory is developed in [19, 20, 21], and is based on work by Fliess and others [22, 23, 24, 25, 26]. We discuss an example in the Methods section.

## Examples from the paper [1]

In its Supplement, the paper [1] presents the following class of systems that have P-invariance, called there “dynamic compensation” (for arbitrary *n*, but the main text specializes to *n* = 1):

$${\dot{x}}_{1}=g\left(p_{2}x_{n+1},x_{n+2}\right)-\mu_{1}x_{1}$$ (7a)

$${\dot{x}}_{i}=\eta_{i-1}x_{i-1}-\mu_{i}x_{i},\;i\in \left\{2,\ldots ,n\right\}$$ (7b)

$${\dot{x}}_{n+1}=x_{n+1}l\left(x_{n+2}\right)$$ (7c)

$${\dot{x}}_{n+2}=k\left(p_{1}x_{1},p_{1}x_{2},\ldots ,p_{1}x_{n},x_{n+2},u\right)$$ (7d)

$$y=x_{n+2}.$$ (7e)

The state variables are positive, that is, $X=R_{>0}^{n+2}$, inputs and outputs are scalar and positive, $U=R_{\geq 0}$, $Y=R_{>0}$, and the parameters for which we desire invariance are two positive numbers, $p=\left(p_{1},p_{2}\right)\in P=R_{>0}^{2}$. The additional parameters *η*_*i*_ and *μ*_*i*_ are fixed, and *g*, *k*, *ℓ* are three scalar (differentiable) functions, with the following positive homogeneity property for *g*:

$$g\left(rx_{n+1},x_{n+2}\right)\;=\;rg\left(x_{n+1},x_{n+2}\right)\;\forall \;r>0,\;x_{n+1}>0,\;x_{n+2}>0\;.$$ (8)

The initial state is implicitly specified in [1] by the requirement that for any parameter vector *p* = (*p*_1_, *p*_2_) there is a unique steady state when *u* = 0; we call this state *ξ*_*p*_. We take the reference parameter set to be 1 = (1, 1).

In the paper [1], when *n* = 1 this system is motivated as a mechanism for a hormonal circuit in which the output *y* is a regulated variable, *x*_1_ is a hormone that regulates *y* = *x*_3_, and the variable *x*_*n*+1_ = *x*_2_ represents the functional mass of the tissue that secretes the hormone *x*_1_. There is feedback on the regulated variable *y*, with a gain *p*_1_, and a growth rate in *x*_*n*+1_ that is controlled by *y*, for instance through increase of proliferation. The parameter *p*_2_ quantifies the magnitude of the effect of the mass on the hormone production rate. In one of the examples in [1], the input *u*(*t*) is the meal intake of glucose, *x*_*n*+1_ represents *β* cells, *x*_1_ is insulin, and *y* is plasma glucose concentration; the coefficients *η*_*i*_ correspond to transport rates, and the *μ*_*i*_ combine degradation rate and transport rates. In the more general case *n* > 1, the coordinates *x*_1_, …, *x*_*n*_ represent the various physiological compartments that the hormone may circulate through.

We show P-invariance by exhibiting an equivariance. In fact, the proof of dynamical compensation in [1] is based (without using that terminology) on showing that the following mapping is an equivariance family:

$$\rho_{p}\left(x_{1},x_{2},\ldots ,x_{n},x_{n+1},x_{n+2}\right)\;:=\;\left(p_{1}^{-1}x_{1},p_{1}^{-1}x_{2},\ldots ,p_{1}^{-1}x_{n},p_{1}^{-1}p_{2}^{-1}x_{n+1},x_{n+2}\right)\;.$$

Since *h*(*x*, *u*, *p*) = *x*_*n*+2_ is not changed by *ρ*_*p*_, we have that *h*(*ρ*_*p*_(*x*), *u*, *p*) = *h*(*x*, *u*), and since as equivariances always map steady states into steady states, and steady states are assumed to be unique, there is no need to test the condition *ρ*_*p*_(*ξ*) = *ξ*_*p*_. So we only need to check that:

$$f(\rho_{p}\left(x\right),u,p)\;=\;{\left(\rho_{p}\right)}_{*}\left(x\right)f(x,u)$$

for all *p*, *x*, *u*. The map *ρ*_*p*_ is linear, and its Jacobian matrix is

$$\text{diag}\;(p_{1}^{-1},p_{1}^{-1},\ldots ,p_{1}^{-1},p_{1}^{-1}p_{2}^{-1},1)\;,$$

thus we need to verify:

$$\begin{array}{rrr} g(p_{2}(p_{1}^{-1}p_{2}^{-1}x_{n+1}),x_{n+2})-\mu_{1}(p_{1}^{-1}x_{1}) & = & p_{1}^{-1}\left[g(x_{n+1},x_{n+2})-\mu_{1}x_{1}\right]\\ \left(i\in \{2,\ldots ,n\})\;\eta_{i-1}(p_{1}^{-1}x_{i-1})-\mu_{i}(p_{1}^{-1}x_{i}\right) & = & p_{1}^{-1}\left[\eta_{i-1}x_{i-1}-\mu_{i}x_{i}\right]\\ \left(p_{1}^{-1}p_{2}^{-1}x_{n+1})l(x_{n+2}\right) & = & p_{1}^{-1}p_{2}^{-1}\left[x_{n+1}l(x_{n+2})\right]\\ k(p_{1}(p_{1}^{-1}x_{1}),p_{1}(p_{1}^{-1}x_{2}),\ldots ,p_{1}(p_{1}^{-1}x_{n}),x_{n+2},u) & = & k(x_{1},x_{2},\ldots ,x_{n},x_{n+2},u), \end{array}$$

the last *n* + 1 of which are trivial, and the first one requires $g\left(p_{1}^{-1}x_{n+1},x_{n+2}\right)=p_{1}^{-1}g\left(x_{n+1},x_{n+2}\right)$, which holds because of the homogeneity property Eq (8).

We next discuss the “linear integral feedback” and “linear proportional integral feedback” examples in the paper [1], which are given as examples of systems that are not P-invariant (the paper does this by simulation). The first system is:

$${\dot{x}}_{1}=x_{2}-b$$ (9a)

$${\dot{x}}_{2}=a-p_{1}x_{1}-p_{2}x_{2}+u$$ (9b)

$$y=x_{2}$$ (9c)

with the unique steady state *ξ*_*p*_ = ((*a* − *p*_2_*b*)/*p*_1_, *b*) for *u*(0) = 0. Let us show that there is no possible equivariance *ρ*_*p*_(*x*_1_, *x*_2_) = (*α*_*p*_(*x*_1_, *x*_2_), *β*_*p*_(*x*_1_, *x*_2_)). The requirement that *h*(*ρ*_*p*_(*x*), *u*, *p*) = *h*(*x*, *u*) means that *β*_*p*_(*x*_1_, *x*_2_) = *x*_2_, so *ρ*_*p*_(*x*_1_, *x*_2_) = (*α*_*p*_(*x*_1_, *x*_2_), *x*_2_). The PDE *f*(*ρ*_*p*_(*x*), *u*, *p*)) = (*ρ*_*p*_)_*_(*x*)*f*(*x*, *u*) translates into:

$$\left(\begin{array}{c} x_{2}-b\\ a-p_{1}\alpha_{p}\left(x_{1},x_{2}\right)-p_{2}x_{2}+u \end{array}\right)\;=\;\left(\begin{array}{c} \frac{\partial \alpha_{p}}{\partial x_{1}}\left(x_{1},x_{2}\right)\left(x_{2}-b\right)+\frac{\partial \alpha_{p}}{\partial x_{2}}\left(x_{1},x_{2}\right)\left(a-x_{1}-x_{2}+u\right)\\ a-x_{1}-x_{2}+u \end{array}\right)\;.$$

Comparing coefficients of *u* in the first component, we have that $\frac{\partial \alpha_{p}}{\partial x_{2}}\;\equiv \;0$, and this in turn implies that $\frac{\partial \alpha_{p}}{\partial x_{1}}(x_{1},x_{2})\;\equiv \;1$. Thus the only possible choice is a linear function *α*_*p*_(*x*_1_, *x*_2_) = *c* + *x*_1_. On the other hand, comparing second components, we have

$$\alpha_{p}\left(x_{1},x_{2}\right)\;=\;\frac{1}{p_{1}}x_{1}+\frac{1-p_{2}}{p_{1}}x_{2}\;,$$

contradicting this formula. Thus, no possible equivariance exists, even if we only ask one of the parameters to vary. Combined with the necessity result, this implies non-invariance.

Another way to analyze this example is as follows. Let us compute the derivatives *m*_*i*_ ≔ *y*^(*i*)^(0^+^) when applying an input *u*(*t*) that is differentiable for *t* > 0. We have that *m*_0_ = *b*, *m*_1_ = *u*_0_, *m*_2_ = −*p*_2_*u*_0_ + *u*_1_, $m_{3}=\left(p_{2}^{2}-p_{1}\right)u_{0}-p_{2}u_{1}+u_{2}$, where *u*_*i*_ ≔ *u*^(*i*)^(0^+^). Therefore, from the output *y* (and in particular from its derivatives at time zero) we can reconstruct *p*_2_ and *p*_1_, for example using a step function *u* ≡ 1: *u*_0_ = 1 and *u*_*i*_ = 0 for *i* ≥ 1 implies that *p*_2_ = −*m*_2_ and $p_{1}=m_{2}^{2}-m_{3}$. When $m_{2}^{2}\leq m_{3}$ there are no possible positive parameters producing this output, and when $m_{2}^{2}>m_{3}$ there is a unique pair *p*_1_ > 0, *p*_2_ > 0 giving the observed derivatives, but in any case, there is never more than one such pair. This means that the parameter vector *p* is identifiable, which implies that the system is not P-invariant.

Alternatively, using the formalism of Lie derivatives, with

$$g_{0}\;=\;(\begin{array}{c} x_{2}-b\\ a-p_{1}x_{1}-p_{2}x_{2} \end{array})\;,\;g_{1}\;=\;(\begin{array}{c} 0\\ 1 \end{array})\;,\;h\left(x\right)=x_{2}$$ (10)

we can compute

$$L_{g_{1}}L_{g_{0}}h\left(x\right)=-p_{2}\;,\;L_{g_{1}}L_{g_{0}}^{2}h=-p_{1}+p_{2}^{2}\;,$$

and we again see that we can recover the parameters, giving identifiability and hence non-invariance.

The second example of a system that fails invariance in the paper [1] is the “linear proportional integral feedback” given by:

$$\begin{array}{lll} {\dot{x}}_{1} & = & x_{3}-x_{1}\\ {\dot{x}}_{2} & = & x_{3}-b\\ {\dot{x}}_{3} & = & a-p_{2}x_{1}-p_{1}x_{2}+u\\ y & = & x_{3} \end{array}$$

with the unique steady state *ξ_p_* = (*b*, (*a* − *p*_2_*b*)/*p*_1_, b) for *u*(0) = 0. The non-existence of equivariances is proved similarly to the previous example, and is not shown. For simplicity, we only perform the Lie derivative test. One can find

$$L_{g_{1}}L_{g_{0}}^{2}h\left(x\right)=-p_{2}-p_{1}\;,\;L_{g_{1}}L_{g_{0}}^{3}h=p_{2}\;,$$

so once more we can recover the parameters, giving identifiability and hence non-invariance.

Let us now illustrate the method of derivatives with the example in Eq 7, which was already shown to be invariant by the method of equivariances. The equations are, in this case:

$$\begin{array}{lll} {\dot{x}}_{1} & = & p_{2}x_{2}x_{3}-x_{1}\\ {\dot{x}}_{2} & = & x_{2}\left(x_{3}-b\right)\\ {\dot{x}}_{3} & = & a-p_{1}x_{1}x_{3}+u\\ y & = & x_{3} \end{array}$$

where *p* = (*p*_1_, *p*_2_) are the parameters of interest, *a*, *b* are other positive parameters, and the equations evolve in the positive orthant *x*_*i*_ > 0. Using, for clarity, superscripts “(*i*)” to indicate derivative of order *i*, and since *y* = *x*_3_, we have that:

$$y^{\left(1\right)}=a-p_{1}x_{1}y+u$$

and thus, taking derivatives of this expression and substituting the equation for ${\dot{x}}_{1}$:

$$y^{\left(2\right)}=u^{\left(1\right)}+yx_{1}^{2}p_{1}^{2}+\left(-ap_{1}-p_{1}u+p_{1}y\right)x_{1}-x_{2}y^{2}p_{1}p_{2}$$

so, taking again derivatives and substituting the equations for ${\dot{x}}_{1}$ and ${\dot{x}}_{2}$:

$$\begin{array}{l} y^{\left(3\right)}=u^{\left(2\right)}-x_{1}u^{\left(1\right)}p_{1}-yx_{1}^{3}p_{1}^{3}+\left(ap_{1}^{2}+p_{1}^{2}u-3p_{1}^{2}y\right)x_{1}^{2}\\ \;+\left(4p_{1}^{3}x_{2}y^{2}+2ap_{1}+2p_{1}u-p_{1}y\right)x_{1}-x_{2}y^{3}p_{1}p_{2}\\ \;+\left(b+1\right)p_{1}p_{2}x_{2}y^{2}+\left(-3ap_{1}p_{2}-3p_{1}p_{2}u\right)x_{2}y. \end{array}$$

Observe that these are all functions of *t* (argument not shown). We can solve for *x*_1_ and *x*_2_ in terms of *y*^(1)^ and *y*^(2)^, obtaining:

$$x_{1}=\frac{a+u-y^{\left(1\right)}}{p_{1}y}$$

and

$$x_{2}=\frac{u^{\left(1\right)}y+\left(a-y^{\left(1\right)}-y^{\left(2\right)}+u\right)y-y^{\left(1\right)}\left(a+u-y^{\left(1\right)}\right)}{y^{3}p_{1}p_{2}}$$

and, finally, substitute these expressions into the formula for *y*^(3)^, to obtain:

$$\begin{array}{l} y^{\left(3\right)}\;=\;\frac{-y^{3}+(b+y^{\left(1\right)})y^{2}+[(-b-2)y^{\left(1\right)}-y^{\left(2\right)}]y+3y^{\left(1\right)}{)}^{2}}{y^{2}}u\\ \;+\frac{-y^{2}+(b+1)y-3y^{\left(1\right)}}{y}u^{\left(1\right)}+u^{\left(2\right)}\\ \;+[-a+y^{\left(1\right)}+y^{\left(2\right)}]y-{\left(y^{\left(1\right)}\right)}^{2}+(a-b)y^{\left(1\right)}+(a-y^{\left(2\right)})b-y^{\left(2\right)}\\ \;+\frac{\left[(b+2)y^{\left(1\right)}{}^{{}^{2}}+(-ab+4y^{\left(2\right)}-2a)y^{\left(1\right)}-ay^{\left(2\right)}]y+3{\left(y^{\left(1\right)}\right)}^{2}(a-y^{\left(1\right)}\right)}{y^{2}} \end{array}$$

which involves only *y*, *y*^(1)^, and *y*^(2)^ but neither *x*_1_ and *x*_2_ nor the parameters *p*_1_ and *p*_2_. Taking derivatives on both sides, and iterating, this implies, in turn, that higher-order derivatives of *y* can also be expressed in terms of *y*, *y*^(1)^, and *y*^(2)^ and neither of the other state coordinates nor *p*_1_ and *p*_2_. This holds for all *t*, so in particular at *t* = 0. We remark that the expression for *y*^(3)^ can also be obtained, when the system equations happen to be given by polynomials, or even by rational functions, using tools from differential algebra. This is discussed in the Methods section.

In the expressions obtained, *y*^(1)^ and *y*^(2)^ depend on the parameters *p* = (*p*_1_, *p*_2_). However, these expressions did not yet use the initial state constraint. Next we prove that *y*(0), *y*^(1)^(0), and *y*^(2)^(0) are independent of parameters, so the same will be true for all derivatives at *t* = 0, of any order, and therefore Proposition 6 will give invariance. Now, specializing at the equilibrium initial state

$$\xi_{p}\;=\;\left(\frac{a}{p_{1}b},\frac{a}{p_{1}p_{2}b^{2}},b\right)\;,$$

we have that, for any differentiable input, *y*(0) = *b*, *y*^(1)^(0) = *u*(0), and *y*^(2)^(0) = −(*a*/*b*)*u*(0) + *u*^(1)^(0). Thus, indeed neither of these derivatives depends on the parameters *p*, which confirms invariance.

It is interesting to observe that, in this example, there is no invariance to the other parameters, *a* and *b*; in fact, these parameters are identifiable. To see this, let us write, for convenience, *α* ≔ *a*/*b*. Then,

$$y^{\left(2\right)}\left(0\right)\;=\;-\alpha \;u\left(0\right)+u^{\left(1\right)}\left(0\right)$$

allows identification of *α*; for example, using the input *u*(*t*) ≡ 1, *Y*_2,1_ ≔ *y*^(2)^(0) = −*α*, so can recover *α* = −*Y*_2,1_. From

$$y^{\left(3\right)}\left(0\right)\;=\;u^{\left(2\right)}\left(0\right)-\alpha \;u^{\left(1\right)}\left(0\right)+\alpha^{2}\;u\left(0\right)-\alpha \;u\left(0\right)$$

we cannot yet identify *a* and *b* individually. Taking one more derivative:

$$\begin{array}{ll} y^{\left(4\right)}\left(0\right)= & -b\alpha u\left(0\right)+u^{\left(3\right)}\left(0\right)-\alpha u^{\left(2\right)}\left(0\right)-3\frac{u{\left(0\right)}^{2}\alpha }{b}\\ & -\alpha^{3}u\left(0\right)+2\alpha^{2}u\left(0\right)+\alpha u\left(0\right)+\alpha^{2}u^{\left(1\right)}\left(0\right)-\alpha u^{\left(1\right)}\left(0\right) \end{array}$$

and therefore, if the input is *u* ≡ 1, we obtain

$$Y_{4,1}≔y^{\left(4\right)}\left(0\right)\;=\;-b\alpha -3\frac{\alpha }{b}-\alpha^{3}+2\alpha^{2}+\alpha$$

and if the input is *u* ≡ 2, we obtain

$$Y_{4,2}≔y^{\left(4\right)}\left(0\right)\;=\;-2b\alpha -12\frac{\alpha }{b}-2\alpha^{3}+4\alpha^{2}+2\alpha$$

and combining these two results, we conclude that 2*Y*_4,1_ − *Y*_4,2_ = 6*α*/*b*, allowing recovery of the parameter *b* (observe that *α* = *a*/*b* ≠ 0, since the parameters *a* and *b* are both positive).

### Relations to FCD

The papers [2, 3] studied a notion of scale invariance, or more generally invariance to input-field symmetries. We restrict attention here to linear scalings

$$u=\left(u_{1},u_{2},\ldots ,u_{m}\right)\;\mapsto \;p⊙u=\left(p_{1}u_{1},p_{2}u_{2},\ldots ,p_{m}u_{m}\right)\;,$$

although the same ideas apply to more general symmetries as well. The property being studied is the invariance of the response of the following parametrized family of systems:

$$\dot{x}=f(x,p⊙u),\;y=h\left(x,p⊙u\right),\;x\left(0\right)=\xi_{p},$$

where the initial states *ξ*_*p*_ are the (assumed unique) steady states associated to *p* ⊙ *u*(0). This property is also called “fold change detection” (FCD), because the only changes that are detectable are possibly “fold” changes in *u*, not simply scalings such as changes of units. It is clear that this is merely a special case of the current general setup.

## Discussion

The dynamical compensation (DC) property was shown in [1] to play an important role in physiological control systems. Independence of parameters means that complete dynamical responses, which may affect phenotype, are robust to potentially large parameter variations. The DC property was verified for various biological circuits including the dependence of glucose under parameters such as insulin sensitivity, using a mathematical test. We showed how the DC property can be re-interpreted in terms of more classical systems notions, and how effective tests for checking DC are then possible, by using the extensive literature on identifiability. We also drew connections to issues of system equivalence, and showed that the “fold change detection” or “scale invariance” property is another manifestation of the same phenomenon.

We have discussed a number of different techniques for testing identifiability (or lack thereof). Each of these techniques has its advantages and disadvantages. Equivariances amount to groups of symmetry and help uncover interesting connections among states and parameters which may have a mechanistic interpretation. When a system is DC, usually an explicit equivariance is easy to guess and it helps “certify” the property, with far less computation than “input/output” methods. The latter involve far less guessing (and no need to solve a partial differential equation), but on the other hand can have huge computational complexity and are difficult to apply to large systems even when there is an obvious structure (as in the example in Eq (7)). Among input/output methods, Lie derivatives or output derivatives at time zero have different advantages for different problems, and it is hard to compare their relative effectiveness. For Lie derivatives, the dimension of the state space is a major parameter in determining computational complexity, while in the case of output derivatives, it is the order of i/o equations that matters, and these two numbers are related in non-trivial ways [27]. In the Lie method, the number of directional derivatives grows exponentially with the input dimension, even for a fixed total order of output derivative. On the other hand, the output derivative method involves symbolic computations with large rational functions, which can easily exceed the available computational storage.

A rather striking aspect of the relation between DC and identifiability is that, while unidentifiability in the context of systems identification is viewed as a “bad” property, meaning that a model is overparametrized or that it is impossible to design experiments to gather enough information about a parameter, in contrast in the context of “dynamical compensation” it becomes a desirable property, as it means that system behavior is robust to changes in these parameters.

It is the case sometimes that even if system behavior theoretically depends on a given parameter, this dependence may be weak. A textbook example is that of an enzymatic reaction operating at near-saturation, where exact concentrations of substrates, if thought of as parameters, may have little influence. Another classical example is furnished by proteases acting in a close to zeroth-order regime. This notion of “almost independence” is also studied in the identification literature, under names such as “practical identifiability” (or more precisely, lack thereof) and is closely related to (though not exactly the same as) the concept of “sloppy” models in systems biology [28, 29, 30, 14, 15, 31]. When applied in the “dynamical compensation” context, these notions should be useful in understanding weaker notions of almost-robustness of homeostatic control systems in physiology.

## Methods

### Proofs of main characterizations

#### Proof of Proposition 1

Fix a parameter *p* and an input *u*(⋅); we must show that Eq (4) is satisfied. Let *x*(*t*) = *φ*(*t*, *ξ*, *u*), *t* ≥ 0, be the solution of the initial-value problem $\dot{x}=f(x,u)$, *x*(0) = *ξ*, so that *ψ*(*t*, *ξ*, *u*) = *h*(*x*(*t*), *u*(*t*)). Viewing the mapping *x* ↦ *ρ*_*p*_(*x*) as a change of variables, we define *z*(*t*) ≔ *ρ*_*p*_(*x*(*t*)). Differentiating with respect to *t*,

$$\dot{z}\left(t\right)\;=\;{\left(\rho_{p}\right)}_{*}\left(x\left(t\right)\right)\dot{x}\left(t\right)\;=\;{\left(\rho_{p}\right)}_{*}\left(x\left(t\right)\right)f\left(x\left(t\right),u\left(t\right)\right)\;=\;f\left(\rho_{p}\left(x\left(t\right)\right),u\left(t\right),p\right)\;=\;f\left(z\left(t\right),u\left(t\right),p\right)$$

where we used Eq (5a) applied with *x* = *x*(*t*) and *u* = *u*(*t*). Moreover, *z*(0) = *ρ*_*p*_(*x*(0)) = *ρ*_*p*_(*ξ*) = *ξ*_*p*_, because of Eq (5c). Thus, since the solution of the initial-value problem $\dot{x}=f(x,u,p)$, *x*(0) = *ξ*_*p*_ is unique, it follows that *z*(*t*) = *φ*(*t*, *ξ*_*p*_, *u*, *p*). Now, by definition,

$$\psi \left(t,\xi_{p},u,p\right)\;=\;h\left(\varphi \left(t,\xi_{p},u,p\right),u\left(t\right),p\right)\;=\;h\left(z\left(t\right),u\left(t\right),p\right)\;=\;h\left(\rho_{p}\left(x\left(t\right)\right),u\left(t\right),p\right)\;=\;h\left(x\left(t\right),u\left(t\right)\right)$$

where the last equality follows from Eq (5b). On the other hand, since *ψ*(*t*, *ξ*, *u*) = *h*(*x*(*t*), *u*(*t*)), we conclude that Eq (4) holds, as desired.

#### Proof of Proposition 3

Consider a piecewise constant control which is equal to *u*^1^ on [0, *t*_1_), equal to *u*^2^ on [*t*_1_, *t*_1_ + *t*_2_), …, and equal to *u*^*k*^ on [*t*_1_ + … + *t*_*k*−1_, *t*_1_ + … + *t*_*k*_), starting from *x*(0) = *ξ*_*p*_. By invariance, the resulting output at time *t* = *t*_1_ + … + *t*_*k*_ is independent of the parameters. Let us denote the *j*th coordinate of this output value as

$$h_{j}\left(t_{1},t_{2},\ldots ,t_{k},u^{1},u^{2},\ldots ,u^{k}\right)\;.$$

It follows that the derivatives with respect to the *t*_*i*_’s of this output are also independent of parameters, for every such piecewise constant control. By induction, one has

$$\frac{\partial^{k}}{\partial t_{1}\ldots \partial t_{k}}{|}_{t_{1}=t_{2}=\ldots t_{k}=0}h_{j}\left(t_{1},t_{2},\ldots ,t_{k},u^{1},u^{2},\ldots ,u^{k}\right)\;=\;L_{X_{1}}L_{X_{2}}\ldots L_{X_{k}}h_{j}\left(x\right)\;,$$

where we are using the notations $X_{l}\left(x\right)=g_{0}\left(x\right)+\sum_{i=1}^{m}u_{i}^{l}g_{i}\left(x\right)$. This expression is a multilinear function of the $u_{i}^{l}$’s, and a further derivation with respect to these control value coordinates gives the elementary observables, which therefore must also be independent of parameters. See [17], Remark 6.4.2 for more details and references.

#### Proof of Proposition 5

If the output is independent of parameters, so are all its derivatives, and in particular the derivatives evaluated at time *t* = 0 are independent of parameters.

We discuss next the notions of accessibility and observability required for the validity of the converse to Proposition 1.

### Accessibility

We consider systems:

$$\dot{x}=f(x,u,p)$$

with the technical condition that *f* is a real-analytic function of *x*. (Outputs do not matter for this section.)

For any fixed parameter vector p∈P, this system is said to be *accessible* from the initial state *ξ*_*p*_ if the set of all states reached from *ξ*_*p*_,

$$R\left(\xi_{p}\right)\;≔\;\left\{x\left(t\right)=\varphi \left(t,\xi_{p},u,p\right),t\geq 0,u\;\text{ranging}\;\text{over}\;\text{all}\;\text{possible}\;\text{inputs}\right\}\;,$$

has a non-empty interior. In other words, $R(\xi_{p})$ cannot be “too thin” (as would happen if there are conservation laws among the species *x*_*i*_, in which case one should first reduce the model to independent variables). See for instance the textbooks [32, 17] for details.

Accessibility can be tested as follows. The Lie bracket of two smooth vector functions $X\to R^{n}$ (more generally, one may extend to vector fields on a smooth manifold, but we restrict here to Euclidean spaces) is the function $[f,g]:X\to {\mathbb{R}}^{n}$ defined by the following formula:

$$\left[f,g\right]\;≔\;\frac{\partial g}{\partial x}f-\frac{\partial f}{\partial x}g\;.$$

Let F={f(·,u,p),u∈U}, that is, the set of vector fields obtained when we plug-in an arbitrary constant input (still for our fixed parameter vector *p*). We now define the set $F_{\infty }$ that consists of all possible iterated brackets [*f*_*ℓ*_, …, [*f*_3_, [*f*_2_, *f*_1_] …]], over all choices of *f*_*i*_’s in F and all *ℓ* ≥ 1. Formally, $F_{\infty }$ is the union of the sets $F_{k}$ that are recursively defined as follows:

$$F_{0}≔F\;,\;F_{k+1}≔\left\{\left[f,g\right]\;|\;f\in F_{k},g\in F\right\}\;,\;k=0,1,2,\ldots .$$

The *accessibility (or controllability) rank condition* is said to hold at *ξ*_*p*_ if there are *n* (the dimension of the state space X) linearly independent vectors among all these vectors when evaluated at the initial state. An equivalent way to state this condition is in terms of the accessibility Lie algebra of the system, which is the Lie algebra $F_{\text{LA}}$ of vector fields generated by F, which is the linear span of $F_{\infty }$, the smallest Lie algebra of vector fields which contains F. The condition is that $F_{\text{LA}}\left(\xi_{p}\right)=R^{n}$, that is to say, the evaluations of these vector fields at the initial state span the entire tangent space. It can be shown that, for affine systems as in Eq (6),

$$F_{\text{LA}}\left(\xi_{p}\right)={\left\{g_{0},\ldots ,g_{m}\right\}}_{\text{LA}}\left(\xi_{p}\right)\;.$$

As an illustration, take the example

$${\dot{x}}_{1}=\alpha u-\delta x_{1}\;,\;{\dot{x}}_{2}=\beta u-\gamma x_{1}x_{2}\;,\;y=x_{2}$$

considered earlier. This is a system affine in inputs, with

$$g_{0}\;=\;(\begin{array}{c} -\delta x_{1}\\ -\gamma x_{1}x_{2} \end{array})\;,\;\;g_{1}\;=\;(\begin{array}{c} \alpha \\ \beta \end{array})\;,$$

and we may compute:

$$\left[g_{0},g_{1}\right]\;=\;(\begin{array}{c} \alpha \delta \\ \alpha \gamma x_{2}+\beta \gamma x_{1} \end{array})\;,\;\;[g_{1},\left[g_{0},g_{1}\right]\;=\;(\begin{array}{c} 0\\ 2\alpha \beta \gamma \end{array})\;.$$

Since the determinant of the matrix with columns *g*_1_ and [*g*_1_,[*g*_0_, *g*_1_]] is equal to 2*α*^2^*βγ* ≠ 0 for every *x*, the accessibility rank condition holds, in particular, at the initial state.

As a second illustration, consider the linear integral feedback system in Eq (9). With the vector fields in Eq (10), we have that *g*_1_ = (0, 1)^*T*^ and [*g*_1_, *g*_0_] = (1, −*p*_2_)^*T*^ are linearly independent and hence span $R^{2}$.

### Observability

Consider again systems as in Eq (1):

$$\dot{x}=f(x,u,p)\;,\;y=h\left(x,u,p\right)$$

where *f* and *h* are real-analytic (initial states do not matter for this section). For any fixed parameter vector p∈P, this system is said to be *observable* provided that, for any two distinct states *ξ* and *ξ*′, there is some input function *u* (which might depend on the particular pair of states *ξ* and *ξ*′) such that the temporal responses *ψ*(*t*, *ξ*, *u*) and *ψ*(*t*, *ξ*′, *u*) differ at at least one time *t*. The equivalent contrapositive of this statement is “*ψ*(*t*, *ξ*, *u*) = *ψ*(*t*, *ξ*′, *u*) for all *u*, *t* implies *ξ* = *ξ*′.” For an analytic input-affine system (6) with output *h* = (*h*_1_,…, *h*_*p*_), one can restate the observability property as follows. We consider the set H of elementary observables $L_{g_{i_{1}}}\ldots L_{g_{i_{k}}}h_{j}$. Two states *ξ* and *ξ*′ are said to be *separated by* observables if there exists some k∈H such that *k*(*ξ*) ≠ *k*(*ξ*′). Observability is equivalent to the property that any distinct two states can be separated by observables, or, in more intuitive terms, that all coordinates of the state can be obtained as (possibly nonlinear) functions of observables. This is analogous to the similar result for parameter identifiability, and its proof is also based on approximating arbitrary inputs by piecewise constant inputs, combining Proposition 2.8.2 and Proposition 6.1.11 in [17] (see also Section 4 in [33]).

As an illustration, take once more

$${\dot{x}}_{1}=\alpha u-\delta x_{1}\;,\;{\dot{x}}_{2}=\beta u-\gamma x_{1}x_{2}\;,\;y=x_{2}$$

We need to show that the coordinates of the states can be recovered from the elements in H. Since *x*_2_ = *h*(*x*), so only need to give a formula for *x*_2_. Using

$$h_{1}\left(x\right):=L_{g_{0}}h\left(x\right)\;=\;-\gamma x_{1}x_{2}\;\text{and}\;h_{2}\left(x\right):=L_{g_{1}}L_{g_{0}}h\left(x\right)\;=\;-\alpha \gamma x_{2}-\beta \gamma x_{1}\;,$$

$x_{1}=-\frac{h_{1}\left(x\right)}{\gamma h(x)}$, or, if we want to avoid division by zero when *y* = 0, $x_{1}=-\frac{h_{2}+\alpha \gamma h\left(x\right)}{\beta \gamma }$.

### Converses under irreducibility and/or analyticity

A system is said to be *irreducible* (alternative terminologies are “minimal” or “canonical”) if it is accessible from the initial state *ξ*_*p*_, and observable.

#### Proof of Proposition 2

The converse of Proposition 1 is entirely analogous to the proof of Theorem 1 in [2] for the special case of FCD, and relies upon the theory of minimal realizations of nonlinear systems. We outline it next. Let us fix any parameter p∈P. We must find a differentiable mapping $\rho_{p}:X\to X$ so that *ρ*_*p*_(*ξ*) = *ξ*_*p*_ and *f*(*ρ*_*p*_(*x*), *u*, *p*) = (*ρ*_*p*_)_*_(*x*)*f*(*x*, *u*), *h*(*ρ*_*p*_(*x*), *u*, *p*) = *h*(*x*, *u*) for all x∈X and u∈U, This means that *ρ* should be an isomorphism [34] between the two systems (1) and (3). Now, Theorem 5 in [34] shows that for two analytic and irreducible systems, I/O equivalence implies the existence of an isomorphism, which is exactly what we require. See [2] for more discussion and details.

#### Proof of Proposition 4

The proof of this converse fact consists two parts: (1) showing that outputs coincide for piecewise constant inputs, which is true because *h*_*j*_(*t*_1_, *t*_2_,…, *t*_*k*_, *u*^1^, *u*^2^,…, *u*^*k*^) can be expressed as a power series in terms of the observables, followed by (2) an approximation of arbitrary inputs by step ones. This step follows, applied to parameter identification instead of state observability, by combining Proposition 2.8.2 with Proposition 6.1.11 in [17]; see also Section 4 in [33], and [21]).

#### Proof of Proposition 6

The proof is analogous to that of Proposition 4. We first consider inputs which are polynomial functions of time. Equality of output derivatives when using polynomial inputs is equivalent to equality using arbitrary differentiable inputs, since as remarked output derivatives are polynomial functions of the inputs and a finite number of their derivatives. Polynomials are in particular analytic inputs, and the system being analytic, the corresponding output functions *y*(*t*) are analytic in *t* (see e.g. the differential equations appendix in [17]). If two analytic outputs have the same derivatives of all orders at *t* = 0, then they are identical (Taylor expansions and principle of analytic continuation) Therefore, for any two parameters, the responses to all polynomial inputs coincide, and therefore one obtains equality for arbitrary inputs, again by an approximation argument as in Proposition 6.1.11 in [17], see also Section 4 in [33] and [21]).

### Differential algebra computation of example

We show here the code needed in order to compute a differential equation for the output derivatives for the example described by Eq (7). Using the package Maple (Maplesoft, Waterloo Maple Inc.), one may employ the command

RosenfeldGroebner(sys,R)

to compute a representation of the radical of the differential ideal generated by a set of equations, in our case (from the equations defining the system)

S ≔ [diff(x1(t), t)-p_2*x2(t)*x3(t)+x1(t), \

diff(x2(t), t)-x2(t)*(x3(t)-b), \

diff(x3(t), t)-a+p_1*x1(t)*x3(t)-u(t), \

y(t)-x3(t)]

and R is a differential polynomial ring, in our case

R ≔ DifferentialRing(blocks = [x1, x2, x3, y, u], \

derivations = [t], arbitrary = [p_1, p_2, a, b])

in which we list the state variables, output variable, and input (in that order), followed by the time variable and the parameters. The command

G ≔ RosenfeldGroebner(S, R)

then computes the ideal, and a final elimination step

M ≔ Equations(G[1], solved)

provides a vector M whose coordinates are, respectively, the expressions for *x*_1_, *x*_2_, *x*_3_ = *y* worked out in the paper, as well as the expression for *y*^(3)^.
